# Evaluation of dexmedetomidine in combination with sufentanil or butorphanol for postoperative analgesia in patients undergoing laparoscopic resection of gastrointestinal tumors: A quasi-experimental trial

**DOI:** 10.1097/MD.0000000000005604

**Published:** 2016-12-16

**Authors:** Xue-Kang Zhang, Qiu-Hong Chen, Wen-Xiang Wang, Qian Hu

**Affiliations:** a Department of Anesthesiology, The First Affiliated Hospital of Nanchang University; b Grade 2015 of Medical Department of Graduate School; c Grade 2014 of Medical Department of Graduate School, Nanchang University, Jiangxi, P.R. China.

**Keywords:** butorphanol, dexmedetomidine, gastrointestinal tumor, postoperative analgesia, sufentanil

## Abstract

The aim of this study was to evaluate the efficacy of dexmedetomidine in combination with sufentanil or butorphanol for postoperative analgesia in patients undergoing laparoscopic resection of a gastrointestinal tumor.

This quasi-experimental trial was conducted in Nanchang, China, from January 2014 to December 2015. Eighty patients (age 27–70 years, American Society of Anesthesiologists physical status I–II) undergoing laparoscopic resection of a gastrointestinal tumor were randomized into 4 groups and offered intravenous patient-controlled analgesia for pain control after surgery. The patients received sufentanil 2.0 μg/kg in combination with dexmedetomidine 1.5 μg/kg (group S_1_) or 2.0 μg/kg (group S_2_), or butorphanol 0.15 mg/kg in combination with dexmedetomidine 1.5 0 μg/kg (group N_1_) or 2.0 μg/kg (group N_2_). Oxygen saturation, mean arterial pressure (MAP), heart rate, visual analog scale score, and Ramsay sedation score were recorded at enrollment (T_0_), at extubation (T_1_), and 4 (T_2_), 8 (T_3_), 12 (T_4_), 24 (T_5_), and 48 (T_6_) hours thereafter. Side effects and satisfaction scores were evaluated after surgery.

MAP increased in all groups at T_1_ but not significantly so when compared with T_0_. Heart rate decreased significantly in group S_2_ when compared with the other groups at T_1_–T_5_ (*P* < 0.05). MAP decreased significantly in group S_2_ when compared with group S_1_ at T_4_–T_6_ (*P* < 0.05). MAP increased significantly in group N_1_ when compared with group N_2_ at T_4_–T_5_ (*P* < 0.05). There was a statistically significant decrease in mean visual analog scale score in group S_2_ when compared with group S_1_ at T_2_ (*P* < 0.05) and group N_2_ at T_1_–T_2_ (*P* < 0.05). Two patients in group S_1_ had vomiting. There were no reports of drowsiness, respiratory depression, or other complications. The satisfaction score was higher in group S_2_ than in the other groups.

Dexmedetomidine in combination with sufentanil or butorphanol can be used safely and effectively for postoperative analgesia in patients undergoing laparoscopic resection of a gastrointestinal tumor. The combination of dexmedetomidine 2.0 μg/kg and sufentanil is particularly beneficial in these patients.

## 1 Introduction

Sufentanil and butorphanol are often used for postoperative analgesia. Sufentanil alone is more likely to cause side effects and respiratory depression than when combined with 1 or more adjunctive drugs in intravenous patient-controlled analgesia (PCA).^[1,2]^ Further, it has been reported that lower doses of butorphanol may have ceiling effects.^[3]^ Effective postoperative analgesia would not only improve patient satisfaction but also reduce the incidence of postoperative complications and shorten the duration of hospitalization.^[4,5]^ According to the available protocols for postoperative pain management, the ideal method is a combination of drugs. Previous studies have reported that use of an α_2_-adrenoceptor agonist can decrease the risk of cardiovascular side effects postoperatively. Dexmedetomidine is a highly selective α_2_ adrenergic receptor agonist with sedative, analgesic, and antianxiety activity.^[6,7]^ However, data on the effects of different concentrations of dexmedetomidine are inadequate. The primary aim of this study was to evaluate the efficacy of dexmedetomidine in combination with sufentanil or butorphanol for postoperative analgesia in patients undergoing laparoscopic resection of a gastrointestinal tumor.

## 2 Methods

### 2.1 Study design and participants

Eighty patients (41 men, 39 women, aged 27–70 years) with American Society of Anesthesiologists physical status I to II and a body mass index < 28 kg/m^2^ undergoing laparoscopic resection of a gastrointestinal tumor were included. The study exclusion criteria included a history of cardiovascular disease, severe renal or hepatic insufficiency, bradycardia, atrioventricular block, chronic pain, and current use of analgesic medication. The patients were randomized into 4 groups of 20 patients each using a computer-generated randomization list to receive dexmedetomidine 1.5 μg/kgand sufentanil 2.0 μg/kg and ondansetron 0.4 mg/kg, diluted with 0.9% saline solution to 100 mL (group S_1_); dexmedetomidine 2.0 μg/kg and sufentanil 2.0 μg/kg and ondansetron 0.4 mg/kg, diluted with 0.9% saline solution to 100 mL (group S_2_); dexmedetomidine 1.5 μg/kg and butorphanol 0.15 mg/kg and ondansetron 0.4 mg/kg, diluted with 0.9% saline solution to 100 mL (group N_1_); and dexmedetomidine 2.0 μg/kg and butorphanol 0.15 mg/kg and ondansetron 0.4 mg/kg, diluted with 0.9% saline solution to 100 mL (group N_2_).

### 2.2 Anesthesia

The patients did not receive any medication before induction of anesthesia. At the start of anesthesia, peripheral venous access was established in the right upper extremity, and a 5-lead electrocardiogram, oxygen saturation (S_p_O_2_), and blood pressure were monitored continuously. Anesthesia was induced by an intravenous infusion of dexmedetomidine 1.0 μg/kg (15 minutes before the start of surgery), etomidate 0.3 mg/kg, sufentanil 0.4 μg/kg, and cisatracurium 0.2 mg/kg. When the trachea was intubated, ventilation was mechanically controlled to maintain a tidal volume of 7 to 10 mL/kg, a respiratory rate of 12 breath/min, and end-tidal carbon dioxide (P_ET_CO_2_) at 35 to 45 mm Hg. Anesthesia was maintained by an intravenous infusion of propofol 4.0 to 8.0 mg/kg/min, remifentanil 4.0 to 8.0 μg/kg/min, and cisatracurium 0.1 mg/kg/min. Hemodynamic stability was maintained intraoperatively. All patients received an intravenous injection of flurbiprofen axetil 50 mg and ondansetron 4 mg 15 minutes before completion of surgery. Propofol, remifentanil, and cisatracurium were then discontinued. All patients were offered an electronic infusion pump for intravenous PCA after surgery.

### 2.3 Outcome measures

Oxygen saturation, mean arterial pressure (MAP), heart rate (HR), visual analog score (VAS), and Ramsay sedation score (RSS) were recorded at enrollment (T_0_), at extubation (T_1_), and at 4 (T_2_), 8 (T_3_), 12 (T_4_), 24 (T_5_), and 48 (T_6_) hours thereafter. Side effects and satisfaction scores were evaluated after surgery.

The VAS score was classified as no pain (score 0), mild pain (score 1–3), moderate pain (score 4–6), or severe pain (score 7–10). Sedation was assessed using the RSS (1, anxious; 2, cooperative and tranquil; 3, responding to command; 4, brisk response to stimulus; 5, sluggish response to stimulus; 6, no response to stimulus).

### 2.4 Ethics statement

The study protocol was approved by the local hospital ethics committee in Nanchang, China, and conducted from January 2014 to December 2015. Informed consent was obtained from all study participants.

### 2.5 Statistical analysis

Normally distributed data are expressed as the mean ± standard deviation. Between-group comparisons were performed using repeated-measures analysis of variance. Categorical variables were compared using the Chi-squared test, and intragroup comparisons were performed using the Wilcoxon rank-sum test. All *P* values < 0.05 were considered to be statistically significant. The statistical analysis was performed using Statistical Package for the Social Sciences version 17.0 software (SPSS Inc., Chicago, IL).

## 3 Results

The patient characteristics are summarized in Table 1. There was no difference in patient sex, age, or weight, or duration of surgery between the study groups (*P* > 0.05). MAP increased in all groups at T_1_ when compared with T_0_, but the difference was not statistically significant (Table 2). HR decreased significantly in group S_2_ at T_1_–T_5_ when compared with the other groups (*P* < 0.05). MAP decreased significantly in group S_2_ when compared with group S_1_ at T_4_–T_6_ (*P* < 0.05) and increased significantly in group N_1_ when compared with group N_2_ at T_4_–T_5_ (*P* < 0.05). A statistically significant decrease in VAS was seen in group S_2_ when compared with group S_1_ at T_2_ (*P* < 0.05) and with group N_2_ at T_1_–T_2_ (*P* < 0.05; Table 3). Two patients in group S_1_ developed vomiting. There were no reports of drowsiness, respiratory depression, or other complications in any of the groups (Table 4). The patient satisfaction rate was higher in group S_2_ (95%) than in the other groups (Table 5).

**Table 1:**
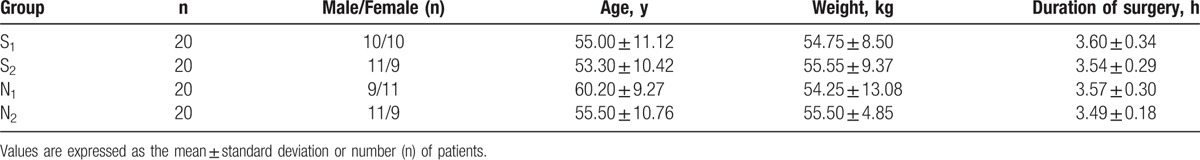
Patient characteristics in the treatment groups.

**Table 2:**
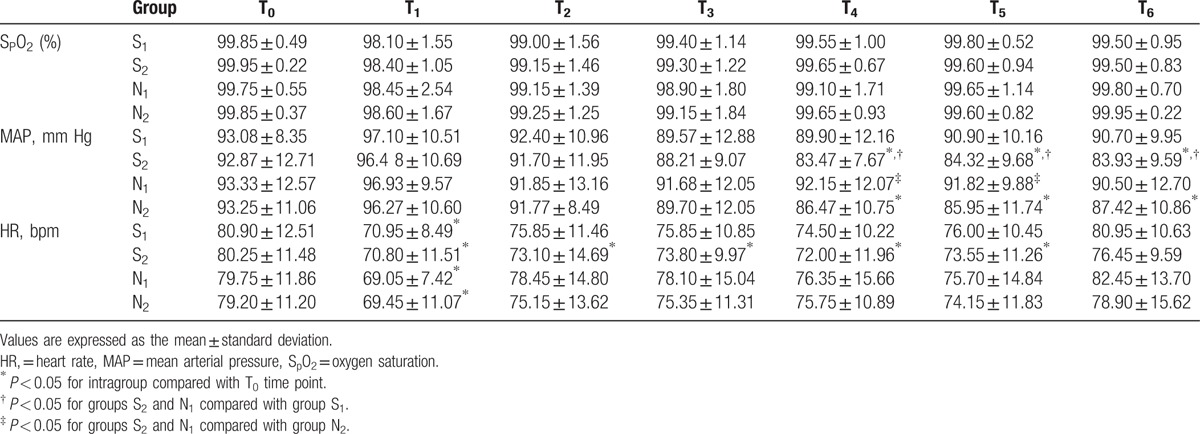
Comparison of hemodynamic changes in the treatment groups at the different time points.

**Table 3:**
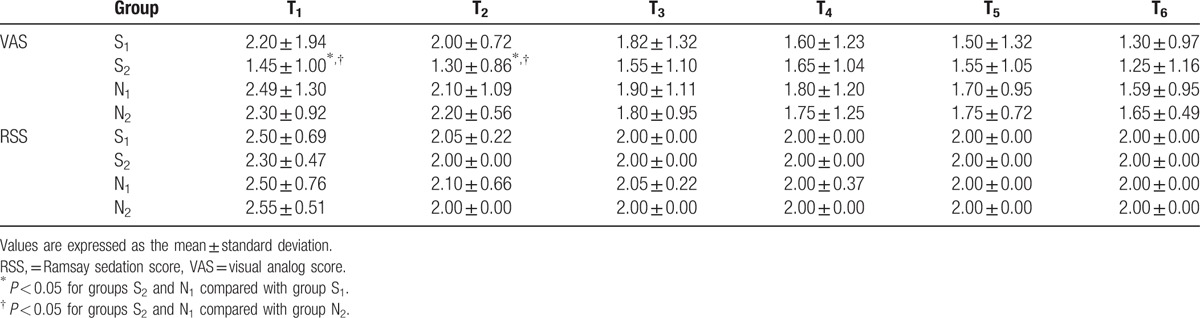
Comparison of visual analog score and Ramsay sedation score between the treatment groups at the different time points.

**Table 4:**
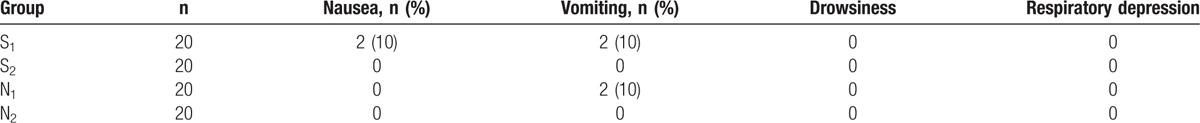
Side effects in the treatment groups.

**Table 5:**
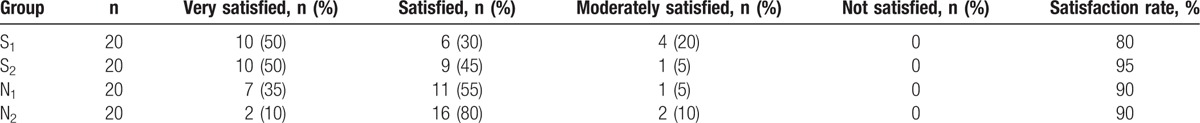
Comparison of patient satisfaction levels between the treatment groups.

## 4 Discussion

Postoperative pain is a common patient complaint after surgery. Apfelbaum et al^[8]^ reported that approximately 80% of their patients experienced pain after surgery and 86% had moderate to severe pain. Although postoperative pain is significantly less after laparoscopy than after open surgery,^[9]^ the pain may still be considerable because of the transabdominal sutures and laparoscopic tacks used during the procedure.^[2,10]^ Medication patches and percutaneous pump devices have been used to decrease postoperative pain with limited success, so improving postoperative analgesia is an area of continued interest in laparoscopic surgery.

The most common type of PCA involves use of an intravenous opioid because of its postoperative analgesic efficacy and prolonged duration of action.^[11]^ However, this type of PCA has considerable side effects, including nausea, vomiting, motor block, urinary retention, and respiratory depression.^[12,13]^ Use of sufentanil has been investigated in some laparoscopic surgery studies. Damen et al^[14]^ reported that intraoperative sufentanil was comparable with remifentanil in patients undergoing laparoscopic cholecystectomy. Early pain was decreased in patients receiving sufentanil, but at the expense of opioid-related adverse effects. Butorphanol is a lipid-soluble narcotic agent with strong κ-receptor agonist and weak μ-receptor agonist/antagonist activity. The above-mentioned narcotic analgesics have been used frequently for postoperative analgesia.^[15]^ In recent years, there have been attempts to reduce the frequency of side effects associated with postoperative pain management by use of a combination of two or more drugs.^[16]^ Recent studies have focused on nonopioid receptors with additional analgesic effects. Previous studies have demonstrated that dexmedetomidine, an α2-adrenoceptor agonist, causes dose-dependent hypotension, bradycardia, and sedation. Dexmedetomidine decreases the HR and blood pressure by decreasing plasma levels of norepinephrine and epinephrine.^[17]^ Saadawy et al^[18]^ reported a decrease in HR and MAP in their dexmedetomidine group within 25 to 35 minutes of caudal administration. In our study, we also found a decrease in HR and MAP, particularly in the group that received dexmedetomidine 2.0 μg/kg. Further, MAP decreased significantly in the group that received dexmedetomidine 2.0 μg/kg and sufentanil 2.0 μg/kg when compared with the other groups. Addition of dexmedetomidine 2.0 μg/kg to sufentanil or butorphanol in this study was not associated with an increased incidence of side effects. Our results are consistent with those of studies in patients undergoing laparoscopic bariatric surgery. In one early study, patients undergoing laparoscopic colorectal surgery who received intraoperative dexmedetomidine reported lower pain scores during the first 12 postoperative hours, and no opioid-sparing effect was found.^[19]^ Dexmedetomidine also showed significant anxiolytic efficacy and durable analgesic efficacy, with a decreased need for postoperative opioid analgesia. Our study found that satisfactory patient sedation contributed to the lessening of postoperative pain. Our results indicated that patients were generally satisfied with their intravenous PCA system because the adjuvant combination of dexmedetomidine and sufentanil or butorphanol therein achieved an acceptable level of sedation.

In conclusion, our results show that the 2 doses of dexmedetomidine as an adjuvant to sufentanil and butorphanol can be safely used for postoperative analgesia in patients undergoing laparoscopic resection of a gastrointestinal tumor. The most effective dose of dexmedetomidine that did not lead to any complications was 2.0 μg/kg combined with sufentanil 2.0 μg/kg.

## Acknowledgment

We would like to express our gratitude to our coworkers and the patients who participated in this study. This study is supported by the National Natural Science Foundation of China (81660096).
